# Datix for Deaths Due to Physical Health Causes Outside Mental Health Inpatient Settings: Staff Perspective

**DOI:** 10.1192/bjo.2025.10361

**Published:** 2025-06-20

**Authors:** Uchechukwu Egbuta, Manorama Bhattarai, Eimear Devlin

**Affiliations:** NHS, Hull, United Kingdom

## Abstract

**Aims:** Datix is a web-based incident reporting and risk management system used across hospitals in United Kingdom to report incidents. DATIX is used primarily for risk management. Therefore the purpose of reporting an incident is to alert the healthcare system to risks and to provide guidance on preventing potential incidents that may lead to avoidable harm or death. Datix can be used to report patient safety incidents or adverse incidents of varying categories such as unexpected effects, medication errors, etc. and these help to provide learning both at individual and organisational level.

The aim is to gather staff perspectives on the current Datix system for deaths secondary to physical health in patients known to mental health settings.

**Methods:** Online Microsoft Form qualitative questionnaire was created to gather staff perspectives on recording of Datix incidents involving deaths due to physical health causes but outside mental health settings. The preliminary questionnaire was shared with Corporate Risk and Compliance Manager, Interim Deputy Director of Nursing in Trust and as advised one of the clinicians attended the Clinical Risk Management Group prior to rolling out to the local Older People’s Community Mental Health Team and Humber Academic programme attendants list. Data was extracted onto Excel for the time March–May 2023 from Microsoft forms. Thematic Data analysis and summary was done collectively by three clinicians in Older Adult.

**Results:** Total: 28 respondents.

Respondent Demographics: approximately 57% nurses; 22% doctors, 7% social workers, 14% team leaders/managers; age 64% below 50 (29% 35–40); 29% 55–65.

7% of respondents have never filled in Datix for death, 36% filled within the last three months.

Source of information: Electronic notes 36%, discussion with colleagues 28%, during review 11%, relatives 14%, never found out 11%.

48% respondents needed to spend a week before finding the cause of death.

Thematic analysis Scale (1 least intensity, 10 highest intensity): Ease of access 14%, In emotionality 43%; Exhausting 61%.

61% respondents did not feel that Datix of deaths caused by physical health needs to be completed by mental health staff. 89% think the process could be made easier.

**Conclusion:** The study shows clearly that most of the respondents did not feel that Datix forms needed to be filled in for older adult psychiatric patients in the community, whose death occurred due to physical health causes but outside mental health setting.

